# The Dynamics of Aerotaxis in a Simple Eukaryotic Model

**DOI:** 10.3389/fcell.2021.720623

**Published:** 2021-11-23

**Authors:** Marta Biondo, Cristina Panuzzo, Shahzad M. Ali, Salvatore Bozzaro, Matteo Osella, Enrico Bracco, Barbara Pergolizzi

**Affiliations:** ^1^Department of Physics, INFN, University of Turin, Turin, Italy; ^2^Department of Clinical and Biological Science, University of Turin, Turin, Italy; ^3^Department of Oncology, University of Turin, Turin, Italy

**Keywords:** aerotaxis, oxidative stress, hydrogen peroxide, collective cell migration, *Dictyostelium*, G-protein, catalase

## Abstract

In aerobic organisms, oxygen is essential for efficient energy production, and it acts as the last acceptor of the mitochondrial electron transport chain and as regulator of gene expression. However, excessive oxygen can lead to production of deleterious reactive oxygen species. Therefore, the directed migration of single cells or cell clumps from hypoxic areas toward a region of optimal oxygen concentration, named aerotaxis, can be considered an adaptive mechanism that plays a major role in biological and pathological processes. One relevant example is the development of O_2_ gradients when tumors grow beyond their vascular supply, leading frequently to metastasis. In higher eukaryotic organisms, aerotaxis has only recently begun to be explored, but genetically amenable model organisms suitable to dissect this process remain an unmet need. In this regard, we sought to assess whether *Dictyostelium* cells, which are an established model for chemotaxis and other motility processes, could sense oxygen gradients and move directionally in their response. By assessing different physical parameters, our findings indicate that both growing and starving *Dictyostelium* cells under hypoxic conditions migrate directionally toward regions of higher O_2_ concentration. This migration is characterized by a specific pattern of cell arrangement. A thickened circular front of high cell density (*corona*) forms in the cell cluster and persistently moves following the oxygen gradient. Cells in the colony center, where hypoxia is more severe, are less motile and display a rounded shape. Aggregation-competent cells forming streams by chemotaxis, when confined under hypoxic conditions, undergo stream or aggregate fragmentation, giving rise to multiple small loose aggregates that coordinately move toward regions of higher O_2_ concentration. By testing a panel of mutants defective in chemotactic signaling, and a catalase-deficient strain, we found that the latter and the pkbR1^*null*^ exhibited altered migration patterns. Our results suggest that in Dictyostelium, like in mammalian cells, an intracellular accumulation of hydrogen peroxide favors the migration toward optimal oxygen concentration. Furthermore, differently from chemotaxis, this oxygen-driven migration is a G protein-independent process.

## Introduction

Oxygen (O_2_) is required for cell survival, oxidative metabolism, and synthesis of adenosine 5′-triphosphate (ATP), being the final electron acceptor in oxidative phosphorylation (Wilson, 2017). Moreover, advances in the understanding of the physiology of O_2_ have shown that it is also important as a signaling molecule. Indeed, in multicellular organisms, it is an essential micro-environmental factor controlling developmental processes (Dunwoodie, 2009).

Maintenance of cellular O_2_ level, supply, and consumption are precisely regulated, as oxygen imbalance could increase reactive oxygen species (ROS) production. To maintain oxygen homeostasis, metazoan organisms use the hypoxic signaling pathway to facilitate O_2_ delivery and cellular adaptation to oxygen deprivation (Claesson-Welsh, 2020). Mammalian cells under hypoxic conditions adapt rather quickly by inhibiting proliferation and relying on glycolysis rather than oxidative phosphorylation for energy production, thus preventing further O_2_ consumption. As a consequence, all these processes increase the production of angiogenic factors that can drive vascular remodeling and eventually improve tissue perfusion and O_2_ delivery (Rey and Semenza, 2010).

The relationship between O_2_ depletion and cancer is a common feature among most solid tumors. The hypoxic core microenvironment provides an aggressive ecological selective pressure for resilient stem-like cancerous cells. Furthermore, hypoxic environmental conditions may induce advanced and dysfunctional vascularization and promote epithelial-to-mesenchymal transition, resulting in high cell mobility and a high risk of developing metastasis (Muz et al., 2015). In this regard, O_2_ gradients are crucial in early stages of sarcoma development, where cells respond to the hypoxic gradient by aggressively invading the matrix, and subsequently show fast and long-distance migration (Lewis et al., 2016). Furthermore, immortalized mammary epithelial cells, residing within deep hypoxia, migrate directionally toward oxygen by the process named aerotaxis, through an ROS-dependent enhanced epidermal growth factor receptor (EGFR) activation (Deygas et al., 2018).

Although the reports described above suggest that O_2_ acts as a chemoattractant for cancer cells to the neighboring blood vessels, the molecular players and signaling pathways triggering this process remain understudied. Aerotaxis has been long studied in prokaryotes (Taylor et al., 1999; Sporer et al., 2017), whereas in eukaryotes it started to be investigated in *Caenorhabditis elegans* (Gray et al., 2004; Chang et al., 2006; Demir et al., 2020), the choanoflagellate *Salpingoeca rosetta* (Kirkegaard et al., 2016), and more recently in mammalian cells (Deygas et al., 2018). In all these cases, cells can first sense and navigate toward oxygen and then react appropriately by eventually organizing a coordinated and directional movement. Nonetheless, the molecular players remain largely unknown.

A relevant part of current knowledge of the mechanisms regulating oriented cell migration, in response to various stimuli, has been obtained in the social amoeba *Dictyostelium discoideum*, which has proven to be an excellent model system to study almost every kind of random and directional cell motility, including chemo-, electro-, and rheo-taxis (Bozzaro, 2013; Lima et al., 2014; Devreotes and Horwitz, 2015; Jeon et al., 2019; Guido et al., 2020). Cell motility is very similar in *Dictyostelium* and neutrophils, and the molecular components involved in cell migration are remarkably conserved between *Dictyostelium* and mammalian cells (Stuelten et al., 2018; Bozzaro, 2019; Pal et al., 2019).

Based on this large plethora of studies and the availability of well-defined mutants in genes regulating cell motility and signal transduction (Fey et al., 2019), we thought that *Dictyostelium* could be a very suitable model for studying aerotaxis. In this report, we analyzed aerotactic migration parameters in *Dictyostelium* wild-type cells and a panel of mutants defective in chemotaxis and/or electrotaxis. In agreement with previous findings in mammals, the oxygen sensing of *Dictyostelium* cells seems very likely associated with high cytoplasmic levels of hydrogen peroxidase. Furthermore, the observed “aerotaxis” does not share the signaling pathways regulating chemotaxis and electrotaxis.

## Materials and Methods

### *Dictyostelium discoideum* Cell Culture and Development

The parental wild-type (WT) AX2 and mutant strains were cultured in an axenic medium (Ashworth and Watts, 1970) at 23°C under shaking at 150 rpm as previously described (Pergolizzi et al., 2017b). The mutant strains were obtained from the Dicty Stock Center^1^, except for mutant HSB1, available in the laboratory, generated by chemical mutagenesis as described (Bozzaro et al., 1987), and previously characterized as temperature-sensitive for the piaA gene (Pergolizzi et al., 2002).

### Aerotaxis Assay

The aerotaxis assay was modified from Deygas et al. (2018). Basically, growing *Dictyostelium* cells were seeded at a density of approximately 10,000 cells/mm^2^ as a 10-μl drop containing 50,000 cells in the center of a 24-well culture plate. After cell attachment to the substratum, 400 μl of the axenic medium was added, and two 14 mm-diameter glass coverslips were placed on top to cover and confine the cell cluster. As a control in the not-confined (NC) system condition, cells were simply seeded without coverslips.

To assay the ability of aggregation competent cells to sense oxygen gradients, axenically growing AX2 cells were washed twice in a Soerensen phosphate buffer and resuspended at 5 × 10^6^ cells/ml. Afterward, a 10-μl drop containing 50,000 cells was spotted in the center of the 24-well culture plate and let adhere for 10 min, before covering with 400 μl of buffer. The cells were then confined immediately (t0) or after 7 h (t7) when the aggregates were well-formed. No confinement (NC) conditions were used as a control of the experiment.

Time lapse movies of cell migration under confinement (C) or no confinement (NC) were recorded with times up to 24 h using a Lumenera Infinity 3 camera coupled to a Zeiss Axiovert 200 microscope, with a 5 × objective. Photograms were taken at intervals varying between 20 and 360 s (Pergolizzi et al., 2017b).

### Quantification of Cell Arrangement During Migration

In our experimental setting, the circular 10-μl *Dictyostelium* cell layer was approximated to a bidimensional system of cells adhering to the substrate.

Therefore, the cell density (i.e., number of cells per unit area) represents a convenient parameter to characterize and define the arrangement of cells within the cluster. To quantitatively measure the density of cells, bright field images were split into portions of a defined and regular area, and the number of cells was determined using the Python package Trackpy (Allan et al., 2021).

Alternatively, in case of high cellular density, a much simpler indirect cell density measurement approach was undertaken represented by the relative number of cells expressed as percentage (%). Briefly, the bright field images were binarized through an adaptive threshold using the Python library OpenCV (Bradski, 2000). As a result, the cells appeared as white shapes on a black background in the binarized images. Brightness, i.e., the fraction of white pixels, can be used to detect changes in cell density as a first approximation. By partitioning the binarized images in circular areas, this method has been used to draw the cell density heat maps and radial profiles of the relative number of cells at different times. The latter were then analyzed to measure the displacement of its peak, which identifies the *corona*.

To investigate the role of cell duplication during collective migration, we instead considered a circular sector of the cell cluster and we segmented single cells using Trackpy (Allan et al., 2021). The increase in the number of cells counted in this specific area can only be attributed to cell duplication, since for symmetry reasons we do not expect on average any net flux of cells through the sector.

### Analysis of the “*Corona*” Features

The most visually striking peculiarity of the migration pattern of the confined *Dictyostelium* cells consisted in the formation of a ring of high cell density, which we named *corona*, that moves away from the hypoxic region.

To characterize the kinetics and pattern features of this process, we first measured the formation time of the *corona* (T^∗^), defined as the time required to detect a local maximum in the cell distribution profile at the periphery of the cell cluster.

Measuring the distance between the peak of cellular density and the center of the colony identified the position of the ring. Next, we determined the kinetics of the ring displacement ΔR(t) (R = radius; t = time) by assessing either its average velocity during the first 2 h after its formation (v_*i*_) or for longer time points (v_*f*_) (i.e., 10–24 h). T^∗^, v_*i*_, and the propagation of the ring have been quantified in at least three experiments for each cellular strain.

The features of the ring dynamics (i.e., width and density in time) were assessed in three independent experiments in the period 10–24 h by setting a minimum threshold value for the cell density. We acquired one measurement every 6 min and performed data binning to group five measurements per data point (data point ± vertical bar = mean value ± standard deviation).

### Estimation of the Expansion Velocity of the Non-confined Cell Cluster

To describe the spreading of the cell cluster in the absence of cell confinement, we analyzed the first 5 h of independent experiments in the non-confined conditions. For each experiment (exp), we located the border of the cluster setting a minimum threshold value (th) for the density profiles; we assessed the displacement of the cluster border to measure the instantaneous expansion velocity of the cluster over 1-min time-lapse [^*exp*^v_*th*_(t)], and we then averaged these values to determine the spreading velocity ^*exp*^v_*th*_. Since the choice of the cell density threshold was arbitrary, we considered a range of possible values, obtaining a set of ^*exp*^v_*th*_ that was averaged to estimate the expansion velocity of the cluster (^*exp*^v). We repeated this evaluation process for three independent experiments, and we measured v_*NC*_ = 0.8 ± 0.3 μm/min (mean value ± standard deviation) that can be used as reference value for further comparisons with the cell dynamics in confined conditions.

### Single Cell Tracking

Employing the Trackpy package, we determined the aerotactic pattern of *Dictyostelium* cells from the point of view of single cells: for both the confined and not confined conditions, we analyzed a video lasting 30 min with a resolution of three frames per minute providing the tracking software with the parameters “typical size” and “maximum speed” of the cells (10 μm and 10 μm/min, respectively). Given the symmetry of the colony, single-cell trajectories were described using polar coordinates (R,θ). The instantaneous speed [| v| (t_*i*_,R)], radial component of the velocity [v_*r*_(t_*i*_,R)], and ratio between them [v_*r*_/| v| (t_*i*_,R)] were measured over time periods of 1 min for each detected cell. These measurements were grouped according to their distance from the center of the colony (R) using the sliding window method (150 μm interval) to describe the average motility of cells in a given position [<| v| >(R), <v_*r*_>(R), and <v_*r*_/| v| >(R)].

Cell tracking was also performed on videos of cells uniformly distributed over a plate to quantify their spontaneous motility in the absence of any hypoxia condition or oxygen gradient.

### Oxygen Level Measurement

Oxygen concentration was determined over time using the VisiSens detector unit DUO1 coupled to the oxygen sensor foil SF-RPSu4. Data were analyzed with the AnalytiCal1 software (PreSens, Germany). Under our experimental conditions, the time lapse of cell displacement and oxygen level detection were two mutually exclusive measurements.

## Results

### *Dictyostelium* Cells Migrate and Regularly Deploy in Response to an Oxygen Gradient

We sought to assess whether *Dictyostelium* cells were able to sense an oxygen gradient and to move directionally toward the oxygen source. To generate an O_2_ gradient, we took benefit of the method previously developed by Deygas et al. (2018) and modified it as indicated in the section “Materials and Methods.” The oxygen gradient was induced by confining (C) the cells under coverslips. As a control, not confined (NC) cells were used. Oxygen levels were directly visualized by dynamic measurements with the VisiSens detector unit, confirming the different hypoxic profiles between the two conditions (Supplementary Figure 1).

**FIGURE 1:**
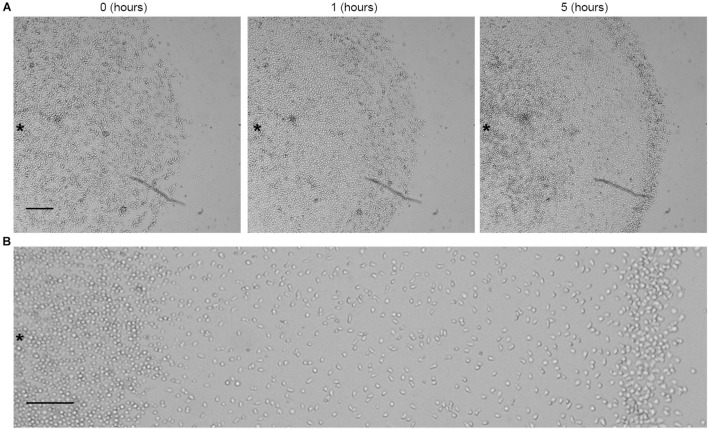
Aerotactic migration of growing AX2 cells. **(A)** Time lapse images of live-cell migration toward a region of higher oxygen concentration (on the right of the image). Time (h) refers to the time that elapsed under confinement. **(B)** Magnification of the image after 5 h of confinement. Scale bar = 200 μm. Asterisks indicate approximately the center of the cell cluster.

A detailed analysis revealed that growing *Dictyostelium* cells moved with a peculiar pattern toward the higher oxygen concentration when subjected to confinement conditions. After approximately 1 h, a fraction of confined cells began to move coordinately, assuming an arrangement characterized by a thickened front, the *corona*, that once shaped, persistently moved toward the oxygen source (Figure 1A and Supplementary Movie 1). Interestingly, underneath the *corona*, the cells lined up in two further regimes that were easily recognized because of their differential cell density (Figure 1B). At the center of the colony, where oxygen concentration was lowest (Supplementary Figure 1), the cells were rather rounded, and their density was highest, although on average it decreases by 35% in the time-range 10–24 h. This stratification of the cluster in three density regimes was found to be fairly constant over time. The central area abutted with a wide region with lowest cellular density, in which the cells displayed an elongated shape (Figure 1B). While the size of the central high-density region was approximately constant, the lowest density sector expanded as the ring moved away from the center. Interestingly, the cells that trigger the formation of the *corona* were not the outermost ones (Supplementary Figure 2 and Supplementary Movies 1, 2).

**FIGURE 2:**
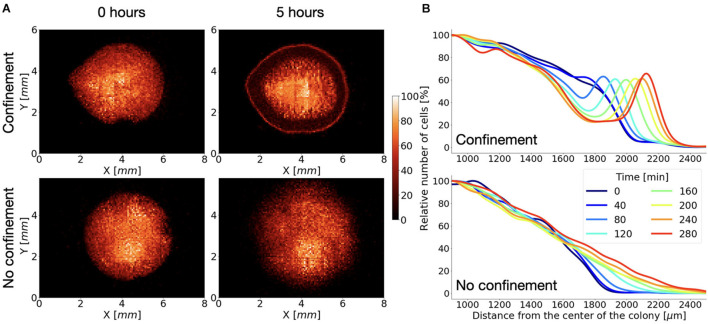
Growing *Dictyostelium* wild-type cells react to the oxygen gradient. **(A)** Distribution of cells at the beginning of the experiment and after 5 h under confined (top) or not confined (bottom) conditions. **(B)** Corresponding cell density profiles (estimated as explained in the section “Materials and Methods”) along the radial direction at different times are shown.

The shape of the cell clusters in C or NC was very similar at the beginning of the experiment (Figure 2A), displaying a circular geometry with the cell density decreasing as it approached the periphery (Figure 2B). Noticeably, in the NC system, cell distribution was maintained throughout the time. The only difference detected was the progressive spreading of the cell cluster with typical velocity of the cluster expansion v_*NC*_ = 0.8 ± 0.3 μm/min, presumably by virtue of random cell motility and cell duplication, as the cells are incubated in a nutrient-rich growth medium.

On the contrary, under confined (C) conditions, we noticed that the pattern of cell arrangement was considerably dynamic over time. When compared with the onset condition, a thickened circular front, the *corona*, featured by a relatively high cell density, emerged, and persistently moved radially following the same direction of the oxygen gradient (Figure 2B).

To exclude that what we observed was the outcome of the pressure caused by the glass coverslips, we also performed the NC experiment by adding an amount of the axenic medium corresponding to the weight of the glass coverslips (0.1 g). Even under such conditions, the cells displayed a behavior identical to that of the NC counterpart (data not shown), indicating that the outcome observed under confinement was tightly associated with hypoxic stress.

### Assessment of the Dynamic Physical Properties of the *corona* Structure

**FIGURE 3:**
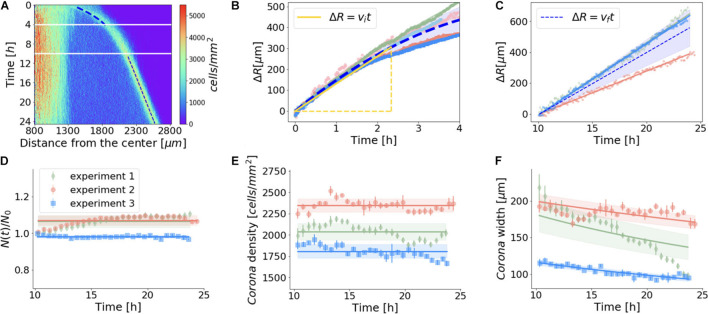
Quantitative description of the aerotactic pattern of growing *Dictyostelium* wild-type cells. **(A)** Cell density for the confined system over 24 h as a function of radial distance and time; blue dashed lines highlight the dynamics of the front in the two time windows that were then studied in detail. Heatmap revealed that over a 24-h time period the very inner core of the cell cluster and the area between the latter and the *corona* maintained two distinct but unaltered density profiles. **(B,C)** Time evolution of *corona* displacement: blue dashed lines represent the average trends [ΔR(t)], computed *via* the mean value of the *corona* displacement measured in different experiments (corresponding to different colors); at any time, the variability of ΔR among the experiments was quantified by the standard deviation, represented by the blue shaded regions. **(B)** Early hours after corona formation: the yellow line shows the time range used to define the initial speed of the front, <v_*i*_> = 2.2 ± 0.2 μm/min (mean value ± standard deviation, measured over five independent experiments). **(C)** Ten hours after confinement, the displacement of the ring grew linearly with time, i.e., with constant velocity v_*f*_. This value displayed large statistical variability among three different experiments, <v_*f*_> = 0.67 ± 0.14 μm/min. In panels **(D–F)**, the continuous lines and the shaded regions correspond to the model of a circular ring of cells that enlarges in radius while keeping the cell density constant without any cell duplication. **(D)** Number of cells over time (estimated as described in Supplementary Figure 3) indicated that cell duplication played a minor role when the ring propagated at constant velocity. Ratio between the number of cells N(t) and the one measured 10 h after the confinement of the colony [N_0_ = N (*t* = 10 h)] was approximately constant. **(E)** Cell density of the *corona* remained constant as the *corona* moves radially. Its value was comparable to the cell density detected in the inner region of the center of the colony. **(F)** Width of the front decreases as it escapes hypoxia.

We then examined the peculiar biological process by which the vegetative *Dictyostelium* cell cluster reacted to the confined system within 24 h. Using density profiles, we determined the time required for the *corona* formation under confinement (T^∗^), which turned out to be nearly 60 ± 20 min for the parental strain AX2 (Supplementary Table 1). Concerning the dynamics of the *corona*, besides experimental variability, we found that after a transient period of a few hours, the initial velocity (v_*i*_) of the *corona* propagation decreased until it reached a constant value (v_*f*_), after approximately 10 h (Figure 3A). The initial velocity (v_*i*_) of the *corona*, measured within the first 140 min, was equal to 2.2 μm/min (Figure 3B), but then it fell sharply to approximately 30% of v_*i*_ (v_*f*_ = 0.67 μm/min) and remained constant for the rest of the experiment, i.e., in the time range 10–24 h (Figure 3C). The decline in the *corona* speed is not sudden but rather smooth. This non-linear velocity trend could simply reflect continuous changes in microenvironmental conditions (e.g., variations of the oxygen profile in the radial direction). Our experimental settings (i.e., cells in the axenic medium and a 24-h time window) are, in principle, compatible with cell proliferation. Indeed, our estimates of cell duplication (Supplementary Figure 3 for details) revealed that, under confinement, the cells were growing within the first few hours, but that after 10 h the number of cells in the colony was essentially constant (Figure 3D). At least within the 10–24 h period, cell growth can be regarded as a negligible effect and, thus, scarcely impacts the *corona* dynamics.

We then assessed the *corona* cell density in the time range 10–24 h. Despite experimental fluctuations, our measurements indicate that, on average, after 10 h, the *corona* cell density reached a steady state (Figure 3E).

**FIGURE 4:**
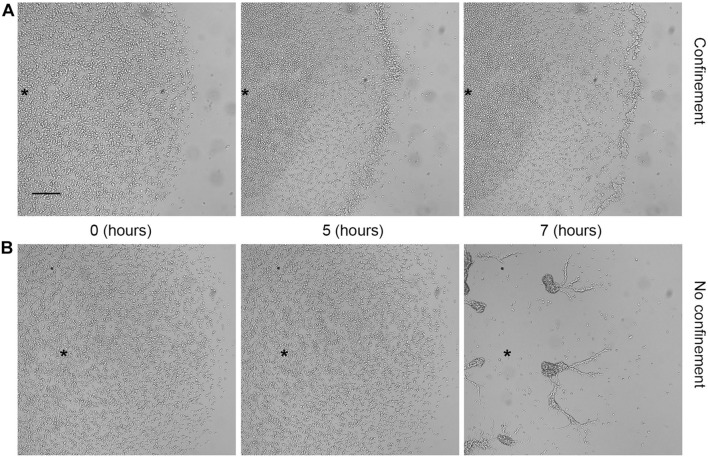
Aerotactic migration occurred independently from cell proliferation. *Dictyostelium* starving cells under **(A)** confinement or **(B)** no confinement conditions. Photograms taken at different times (0, 1, and 5 h) using Zeiss Axiovert 200 microscope, with a magnification of 5×. Scale bar = 200 μm. Asterisks indicate approximately the center of the cell cluster.

If the number of cells that compose the *corona* and coordinately migrate is constant, as suggested by the fact that cell duplication is negligible, in order to maintain the same cell density, the width of the *corona* has to decrease. These simple geometric arguments provide an analytic prediction given the circular shape of the *corona* and the empirical linear growth in time of the *corona* radius R(t) = R0 + ΔR(t). If a *corona* of width L(t) and radius R(t) has to conserve the total area of the ring, its width will decrease as L(t + Δt) = L(t)R(t)/R(t + Δt). The parameters of this model are the average number of cells [N(t)], the cell density within the *corona*, and its area. For each experiment (represented in Figures 3C–F by different colors), we independently estimated the best values of these parameters and their error (mean ± standard deviation), and used them to produce respectively the continuous lines and the shaded regions in Figures 3D–F, which can explain reasonably well the empirical trends that we observed. To further exclude a role of cell duplication in the formation and progression of the *corona*, we assayed aerotaxis in starving WT cells, which are unable to duplicate. Even under these conditions, the cellular behavior was indistinguishable from that of growing cells (Figure 4 and Supplementary Movie 3). As expected, under starvation, the cells lying within the *corona* reached the aggregation competence with timing comparable with that of the unconfined cells. Indeed, a *corona*-like front emerges within 2 h and persists for up to 4–5 h. By this time, the starving cells become aggregation-competent and start aggregating by chemotaxis driven by self-generated cAMP. This leads to fragmentation of the *corona* in streaming aggregates. These results confirm that cell division plays a negligible role in the formation of the *corona*. In addition, this assay proves that the exhaustion of nutrients does not represent a key event in triggering the movement toward the oxygen source, because otherwise the *corona* formation of starving cells would be faster than the response of growing cells. Ultimately, these findings also suggest that the *corona* structure represents a barrier to the oxygen to flow in the inner core of the cluster and when fragmented, because cells aggregate, and the oxygen gradient becomes discontinuous, thus inhibiting the formation of a new *corona*.

### Different Motility Features Are Associated With the Cluster Density Profiles

**FIGURE 5:**
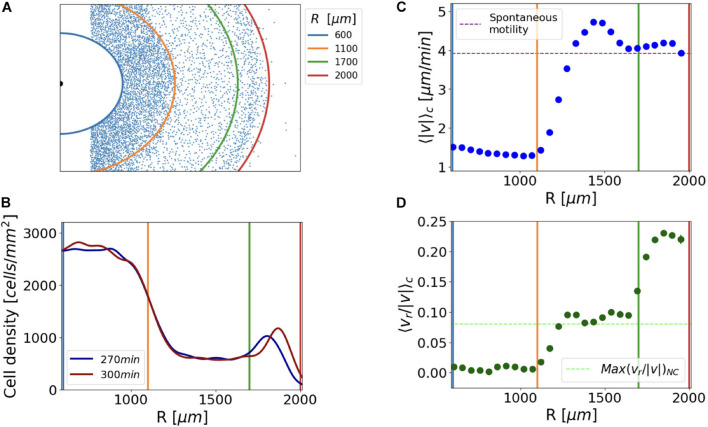
Confined growing *Dictyostelium* cells displayed different motility profiles associated with areas of different cell densities. **(A)** Distribution of the tracked cells; continuous lines delimit three cell density regimes and serve as references for the plots that follow. **(B)** Cell density profiles did not change significantly in the observation time of 30 min, during which we performed cell tracking. **(C)** Cell average instantaneous speed <| v| >_*C*_ as a function of their radial position R; the purple dashed line corresponds to the average speed measured for the WT spontaneous motility assay. **(D)** Average cosine of directions relative to the oxygen gradient direction <v_*r*_/| v| >_*C*_ as a function of their radial position; as a benchmark for the directionality of the cells, we plot the maximum directionality measured in the not-confined cell cluster [Max(<v_*r*_/| v| >_*NC*_) = 0.08], (green dashed line).

We examined single-cell tracks of the confined cluster to ascertain cell motility features, such as average directionality, speed, and their association with the distance from the center. A survey was carried out in three different regions, namely, the center of the cell cluster, the *corona*, and the region in between (Figure 5A). The analysis was performed over 30 min in the time frame between 270 and 300 min, which was a convenient time scale to track thousands of cells per frame without significant changes in the cell cluster density profile and in the *corona* displacement (Figure 5B). To quantify the single cell motility, we measured the average instantaneous speed [<| v| >_*C*_], at different distances from the cluster center. Conversely, the cell directionality was examined, by determining the average ratio between the radial component of the instantaneous velocity and the instantaneous speed [<v_*r*_/| v| >_*C*_]. To assess the average directionality of cells in the absence of a substantial oxygen gradient, we tracked the cells of the not confined system during the same timeframe.

Our analysis identified three different motility profiles, strictly associated with the three regions of different cellular density previously described. On average, cells lying in the center of the cluster (600 μm < R < 1,100 μm) displayed a rather stationary behavior with minor or no displacement (Figure 5C). Notably, those fewer cells showing displacement did not exhibit any preferential direction with respect to the geometry of the system [<v_*r*_/| v| >_*C*_ = 0].

The cells embedded in the *corona* (*R* > 1,700 μm) displayed an average instantaneous speed comparable with that of cellular random motility (3.9 μm/min, Figure 5C). For the sake of clarity, we refer to random motility as that recorded for growing cells seeded under no confinement and incubated in the axenic medium. The comparison between the average <v_*r*_/| v| >_*C*_ of the cells belonging to the *corona* and the directionality measured in the non-confined cell cluster <v_*r*_/| v| >_*NC*_ allowed us to conclude that the former exhibited evident directionality toward regions of higher oxygen concentration. As a matter of fact, the value of <v_*r*_/| v| >_*NC*_ calculated as a function of the distance from the center had a maximum value of 0.08 in the outer colony region. On the other hand, <v_*r*_/| v| >_*C*_ is 0.24 if evaluated in the *corona* returned to 0.08 in the low-density region (Figure 5D). In order to support this result, we performed a Von Mises fit of cell angular displacement distributions to estimate the mean direction (μ) and the accuracy of the orientation (k) of the cells of the *corona* (Fisher and Annesley, 2006). Given the initial cell distribution within the cluster and the circular symmetry of the system, it was not surprising that the mean direction of cells was the radial one. However, we next imposed μ = 0, and assessed the accuracy of the orientation (k). The directionality of the cells belonging to the *corona* was higher than the one measured for the confined cells in the intermediate region and for cells in the not confined cluster (Supplementary Figure 4A).

When compared with the very inner region of the cell cluster, cells in the intermediate low-density region, (1,100 μm < R < 1,700 μm) showed higher speed with a velocity peak immediately behind the *corona* (Figure 5C).

In conclusion, cells in the intermediate region displayed an appreciable movement directionality toward the oxygen source <v_*r*_/| v| >_*C*_ > 0 (Figure 5D), but compatible with the random spreading of *Dictyostelium* from a high cell density region as measured in the not-confined system [<v_*r*_/| v| >_*C*_ ≈ Max(<v_*r*_/| v| >_*NC*_)]. Similarly, the average radial velocity of the cells in the low-density region was compatible with the rate of expansion of the non-confined cluster <v_*r*_>_*C*_ ≈ v_*NC*_ (Supplementary Figures 4B,C) suggesting again that the migration in this area could not be driven by the oxygen gradient.

The reduced directionality of the cells of the intermediate region with respect to those belonging to the *corona* implies that it is unlikely that cells of the intermediate region can supply the *corona*. Therefore, it is likely that the cells that migrate coordinately in the *corona* are always the same (i.e., their number is conserved).

### Hypoxia Selectively Blocks *Dictyostelium* Cell Aggregation and Triggers Oxygen-Dependent Collective Migration

**FIGURE 6:**
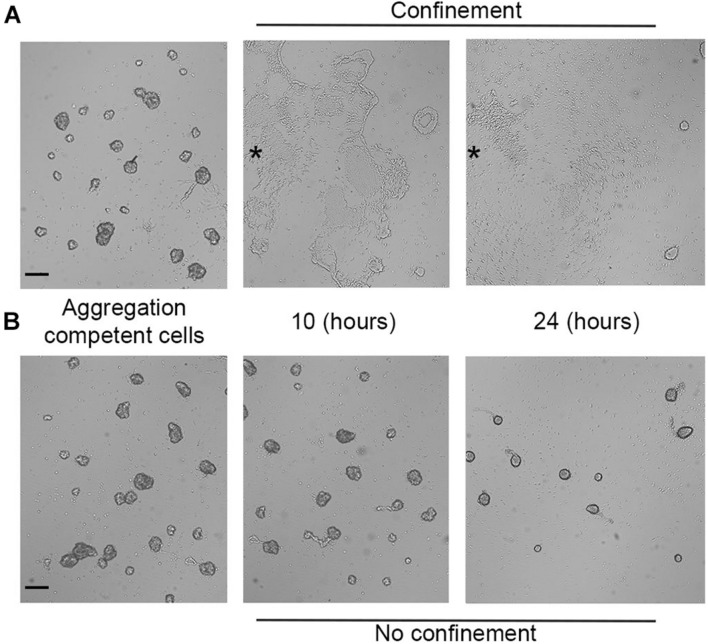
Aerotactic migration of aggregation-competent cells. Photograms taken at different times (7, 10, and 24 h) using a Zeiss Axiovert 200 microscope, with a magnification of 5×. Aggregates under **(A)** confinement or **(B)** no confinement. Scale bar = 200 μm. Asterisks indicate approximately the center of the cell cluster.

When starved under normoxic conditions (18–21% O_2_), *Dictyostelium* cells aggregate, generate migratory slugs, and ultimately culminate to form fruiting bodies. Oxygen concentration regulates the aggregate size and the orientation of the prestalk-prespore pattern (Sternfeld and Bonner, 1977; Sternfeld and David, 1981), while below 10%, oxygen hinders culmination (Sandonà et al., 1995). To test the ability of aggregation-competent cells to react to hypoxia, we assessed aerotaxis in cells starved for 7 h. Briefly, aggregates formed under submerged conditions were confined and then assayed. Within the first few minutes, the aggregates suffered profound rearrangements, getting looser and partially disaggregating. Eventually, within the next 30–40 min, the loose aggregates directionally moved following the oxygen gradient (Supplementary Movie 4). Single cells still exhibited their elongated shape and arranged themselves in streaming-like structures (Figure 6A) that eventually moved toward the oxygen source displaying collective cell migration. Under such conditions, the absence of a clear-cut corona can be a consequence that the aggregates give rise to a discontinuous hypoxic gradient all over the cell cluster.

In the NC control, the aggregates became more compact as time progressed (Figure 6B).

### *Dictyostelium* Aerotactic Migration Relies on Impaired Intracellular Hydrogen Peroxide Degradation but Not on Chemotaxis Signaling Pathways

In mammalian cells migration toward an oxygen gradient is associated with a significant accumulation of H_2_O_2_ at the border of the cell cluster. This represents the most prominent event coinciding with the onset of directional motility (Deygas et al., 2018).

Based on this finding, we sought to assess the aerotaxis process in a *Dictyostelium* mutant defective in catalase activity (Garcia et al., 2002). This enzyme catalyzes the decomposition of H_2_O_2_ into H_2_O and O_2_. As a consequence, mutants with no catalase activity are hypersensitive to oxidative stress from hydrogen peroxide. Indeed, *catA*^*null*^ cells display a severely impaired response to hydrogen peroxide even at a very low concentration (Supplementary Figure 5).

**FIGURE 7:**
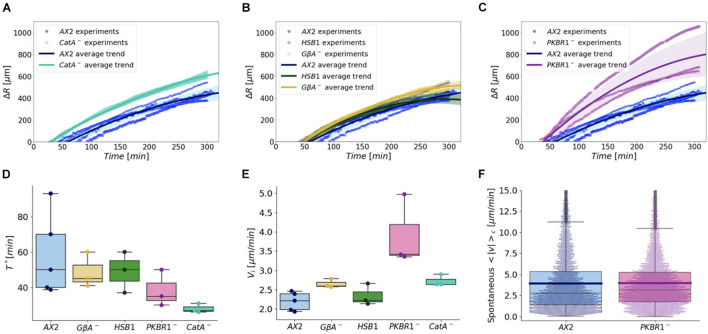
Comparison between the migration of wild-type cells and various chemotaxis deficient mutants under growing conditions. **(A–C)** We report the time evolution of the corona displacement of independent experiments (colored bullets): different colors represent different *Dictyostelium* strains, whereas the size of the markers corresponds to distinct experiments. To group the corona displacement trends of the same genotype, we represented the mean value of all the experiments (continuous colored lines) and their standard deviation (colored shaded regions). Comparison of the ring dynamics **(A)** between WT and *catA*^*null*^ mutants; **(B)** among WT, *HSB1*, and *G*β^*null*^ mutants; **(C)** between WT and *pkbR1*^*null*^mutant. Boxplots in panels **(D,E)** summarize, respectively, the estimated ring formation time T* (measured over at least three experiments for each cell type) and the ring initial propagation speed v_*i*_. **(F)** The spontaneous motility (measured as the average instantaneous speed) under not confined conditions for single cells of the WT and *pkbR1*^*null*^ strains (mean values are represented by the blue and purple horizontal lines).

The aerotactic migration of the *catA*^*null*^ mutant was assayed and compared with that of the WT. With regard to the cell density profiles, they were indistinguishable. On the contrary, the kinetics of the process highlighted that the time required to organize the *corona* (T^∗^) was shortened for *catA*^*null*^ when compared with that of the WT cells (Figure 7A). The *catA*^*null*^ showed a decline of T^∗^ of approximately 50% (28 ± 2) (Figure 7D and Supplementary Table 1). In contrast, the initial velocity (v_*i*_) of the *corona* propagation of *catA*^*null*^ cells exhibited only a moderate increase when compared with that of the WT (Figure 7E and Supplementary Table 1). Therefore, although evolutionarily distant, both *Dictyostelium* and mammalian cells utilize intracellular H_2_O_2_ as an activator or enhancer of aerotactic migration.

We also tested a panel of mutants defective in chemotaxis and/or electrotaxis to assess whether they displayed altered aerotaxis, suggesting potential links between these motility processes. As summarized in Supplementary Table 2, the mutants either fail to aggregate at all or display impaired aggregation because of defects in the signaling pathways. A preliminary qualitative analysis of all the mutants showed that none of the strains displayed major defects in aerotaxis.

Quantitative analysis was restricted to three mutants: the *G*β*^*null*^* mutant, which lacks the beta subunit of the G protein and is, thus, essential for transducing GPCR chemotactic signals (Lilly et al., 1993; Pergolizzi et al., 2017a); the HSB1 mutant, harboring the *pianissimo* gene mutation pia^*G*917*D*^, required for adenylyl cyclase activation (Pergolizzi et al., 2002); and pkbR1^*null*^ (Meili et al., 2000), defective in the PKB/AKT related kinase. Under confinement, all the mutants behave as the WT cells (Figures 7B,C and Supplementary Figure 6). With the only exception of the *pkbR1*^*null*^ strain, in which a moderate decline in the time required for *corona* formation (T^∗^) was observed, for the other mutants, no significant differences were detected (Figure 7D and Supplementary Table 1). However, the *pkbR1*^*null*^ and, to a much lesser extent, *G*β*^*null*^* strains exhibited higher initial propagation speed of the *corona*, and the latter similar to *catA*^*null*^ (Figure 7E and Supplementary Table 1). These results suggest that signals transduced *via* G-protein coupled receptor(s) and PKBR1 serve to restrain or inhibit the sensitivity of the cells to O_2_ gradients in aerotaxis. Conversely, such differences were abolished when considering the time scale of within 10–24 h (Supplementary Figure 7). To rule out that the prominent initial speed detected in *pkbR1*^*null*^ cells was the outcome of increased single cell motility, we also measured its spontaneous motility and compared it with that of the WT. We found that the random movement of *pkbR1*^*null*^cells displayed an average speed basically indistinguishable from that of the WT strain AX2 (Figure 7F).

## Discussion

Mild to severe O_2_ deprivation (hypoxia) due to high tumor cell metabolic rate and aberrant vascularization is associated with altered cellular metabolism as well as resistance to chemotherapy and radiotherapy (Horsman and Overgaard, 2016; Parks et al., 2017). Furthermore, hypoxia regulates cell proliferation and supports apoptosis evasion by tumor cells. On the whole, hypoxia exerts a selection pressure that leads to the survival of subpopulations of viable cells with the genetic machinery for malignant progression. Indeed, hypoxia has been associated with metastasis, which usually leads to a very poor prognostic outcome (Rankin and Giaccia, 2016). Although metastasis accounts for the great majority of cancer-associated deaths, this complex process still remains the least understood aspect of cancer biology (Lambert et al., 2017). In this regard the prerequisites for the migration of tumor cells from a primary site to a secondary/metastatic site include weakening of the cell-to-cell adhesion and capability to directionally move away from the tumor mass toward an oxygenated blood vessel (Seyfried and Huysentruyt, 2013). While the molecular mechanisms responsible for the lessening of the intercellular cell adhesion have been largely ascertained, those enabling the directional “navigation” away from the mass have, only recently, started to be explored.

Besides bacteria (Taylor et al., 1999; Sporer et al., 2017), response to O_2_ gradient has started only lately to be assessed in simple eukaryotic model systems (Gray et al., 2004; Chang et al., 2006; Kirkegaard et al., 2016; Demir et al., 2020) and more recently in mammalian cells (Deygas et al., 2018).

Early studies on *Dictyostelium* demonstrated that the cells responded by moving up the oxygen gradient, while late in development, the prestalk-prespore pattern can be oriented by the gradient (Sternfeld and Bonner, 1977; Sternfeld and David, 1981). Hence, we have attempted to quantitatively assess whether *Dictyostelium* cells can escape hypoxia moving along an oxygen gradient. Our findings indicate that, as in other organisms, *Dictyostelium* cells react to hypoxia in a time scale of 30-60 min. This implies the presence of a very efficient detection mechanism. Our current results do not allow to distinguish between positive (aero)taxis or negative chemotaxis because of accumulation of repellent/s. However, depletion of key nutrients can be excluded, as starving cells showed the same behavior as growing cells. In this context, however, we can maintain that migration is the result of oxygen gradient as a phenomenon, without implying a mechanistic cause-effect.

The cells migrate following the gradient by adopting a peculiar arrangement, consisting of a peripheral structure (*corona*) characterized by contiguous cells exhibiting high speed and significant directionality toward a region of suitable oxygen level. Interestingly, the cells that converge to form the *corona* are not the most external ones, which would be those closer to the region with high oxygen concentration. Instead, the local density maximum, which identifies the *corona*, forms within the colony radius. If the *corona* formation was simply caused by a critical hypoxic level, the migration would, in principle, start from the center of the colony, and all cells would move to escape the region of hypoxia, as long as the critical value does not lead to rounding of the cells. On the other hand, if the trigger was only the presence of an oxygen gradient, the migration would start earlier at the border of the colony, where we measured a slight oxygen gradient already 5 min after the confinement. A minimal model of this phenomenon (excluding intercellular communication) could consider as possible cause of the observed migration a critical hypoxic level in the presence of an oxygen gradient.

In contrast to *Dictyostelium* clusters, mammalian cell clusters under hypoxic conditions do not arrange so neatly, although a sort of “inner-core,” characterized by almost stationary cells, is common to both cell types (Deygas et al., 2018). The mechanism leading to this singular arrangement assumed by *Dictyostelium* cells remains currently unknown. Nevertheless, a detailed analysis of the dynamics of aerotaxis in *Dictyostelium* suggests that such arrangement is not random but that it is dictated by dynamic microenvironmental conditions. Behind the *corona*, displaying a rather high cellular density, we identified a wider region with lower density, in which the cells are appreciably elongated. Remarkably, the cells immediately behind the *corona* showed the highest speed, although their directionality was lower than that of the *corona*. We also noticed that, even if *Dictyostelium* WT cells are incubated in the axenic medium, after approximately 10–15 h, cell duplication is largely inhibited.

While drafting this manuscript, the team of Rieu reported results that mostly overlapped with ours (Cochet-Escartin et al., 2021). However, the experimental settings are quite different, and there are some contrasting results. For example, they suggest that, in growing cells, cell division plays an important role during aerotactic migration, while in our case, if any, this is restricted exclusively to the early-mid events. Noteworthy, our findings achieved by starving cells strengthen the hypothesis that, at least under our experimental settings, the role played by cell division is negligible for reacting to an oxygen gradient and for the *corona* formation and propagation.

Such discrepancy might be due to the different number of cells used for the spot assay to induce the oxygen gradient, giving rise to different microenvironmental conditions. Similarly, their experiments do not show radial motion of the cells outside the *corona*.

Overall, our analysis indicates that the aerotaxis migration differs from all the other *Dictyostelium* taxis (i.e., chemo-, electro- and rheo-taxis) so far described in the social ameba (Zhao et al., 2002; Decave et al., 2003; Lima et al., 2014; Devreotes and Horwitz, 2015; Gao et al., 2015). We also assessed the migration behavior of developing cellular aggregates under confined conditions. Diversely from vegetative cells, aggregating cells have acquired different properties, such as the ability to produce and secrete the chemoattractant cAMP and to tightly adhere to each other by virtue of the adhesion molecule gp80/csA (Noegel et al., 1985). Accordingly, *Dictyostelium* cell aggregates have recently been regarded as a potential and challenging model to investigate solid tumor mass (Trigos et al., 2018; Mathavarajah et al., 2021). Under hypoxic conditions, the aggregates became loose and portions of them detached within minutes. This finding suggests weakening of cell-cell adhesion. Even if different time scales and distinct molecular players are involved, such behavior recalls the epithelial-mesenchymal transition that is pivotal in favoring the metastasis process (Pastushenko and Blanpain, 2019). Our data indicate that, because of the short time window, the reduced cell-cell adhesion is independent from a transcriptional event, suggesting rapid regulation, internalization, or post-translational modification of the adhesion molecules. Furthermore, these findings definitely indicate that hypoxia-sensing mechanisms are present at both the vegetative and aggregation stages, suggesting a common sensor that is retained throughout *Dictyostelium* life cycle. This would be consistent with early reports that an oxygen gradient regulates aggregate size, migration, and pattern formation (Sternfeld and Bonner, 1977; Sternfeld and David, 1981) as well as gene expression (Schiavo and Bisson, 1989; Sandonà et al., 1995).

In animals, a crucial oxygen sensor is the transcription factor HIF1α that under normoxic conditions has a very short half-life, while its degradation is retarded under decreasing oxygen concentration conditions. The more stable HIF1α further translocates to the nucleus, where it dimerizes with HIF1β, and the dimer eventually regulates gene transcription (Semenza, 2012). However, very recent data with animal cells (Kirkegaard et al., 2016; Deygas et al., 2018) indicate that HIF1α does not act as a sensor for aerotaxis. The *Dictyostelium* genome does not encode for the putative HIF1α. Interestingly, in mammalian cells incubated under hypoxic conditions, the driving event triggering the cell movement toward an oxygen source is intracellular accumulation, restricted to the very peripheral cells of the cluster, of hydrogen peroxide (Deygas et al., 2018). Hydrogen peroxide is a rather unstable molecule. However, it is more stable and less noxious than other reactive oxygen species (ROS), but interconvertible with other forms such as hydroxyl radicals and singlet oxygen by iron-catalyzed Haber-Weiss reactions. Like other cells, *Dictyostelium* amebae dispose hydrogen peroxide with the enzyme catalase, degrading it to H_2_O + O_2_ (Parish, 1975; Hayashi and Suga, 1978; Sepasi Tehrani and Moosavi-Movahedi, 2018). The *Dictyostelium* genome harbors two catalase-encoding genes, *catA* and *catB*. Whereas the latter is prespore-specific, the former shows a fairly constant gene expression level at all stages of growth and development (Garcia et al., 2000; Garcia et al., 2002). Hence, we analyzed the aerotactic behavior of the *Dictyostelium catA*/null strain that, although aggregating and developing normally, reacted to oxygen deprivation faster than the WT strain. How can the suppression of catalase A affect cell migration? Hypoxia has been shown to favor the synthesis of plasmalogens, which occurs in peroxisomes (Jain et al., 2020). Their synthesis will increase the ratio of unsaturated vs. saturated phospholipids in the membrane, favoring membrane fluidity (Nagura et al., 2004; Dean and Lodhi, 2018), which in turn could result in increased cell motility (Taraboletti et al., 1989; Edmond et al., 2015; Zhao et al., 2016; Angelucci et al., 2018). We speculate that ROS accumulation, resulting from hypoxia, stimulates plasmalogen synthesis, a process further enhanced by catalase A inactivation. It is worth mentioning, in this context, that the plasmalogen form of phosphatidylethanolamine is the major phospholipid constituent of *Dictyostelium* cell membrane (Weeks and Herring, 1980). In addition, hydrogen peroxide is also a crucial regulator of the cytoskeleton dynamics on the leading edge of migrating cells and thus controlling cell polarity (Hurd et al., 2012; Emre et al., 2021).

Overall, these results let hypothesize that catalase, by regulating intracellular hydrogen peroxide pool, represents a component or regulator of a putative sensor machinery plausibly conserved throughout different kingdoms.

In an attempt to identify other possible players regulating aerotaxis, we tested a panel of mutants defective in chemotaxis. In terms of signal transduction mechanisms, *Dictyostelium* chemotaxis and electrotaxis diverge relatively little (e.g., G-protein and PKBR-1), while most of the players appear to be shared (pianissimo, rip3, and PTEN, etc.). Because we were unable to examine the whole collection of chemo- and electro-taxis mutants, we chose several archetypal strains and analyzed their behavior. *Dictyostelium* cells possess only one Gβ subunit, which is essential for all G protein-dependent signal transduction pathways. Unlike chemotaxis, but more similar to electrotaxis, the G protein appears to be a secondary regulator constraining the bias shown by our survey carried out with the *G*β*^*null*^* mutant. Likewise, our findings indicate that *Dictyostelium* hypoxia-driven migration is a process independent from mTORC2 that is, on the contrary, an essential regulator for both chemo- and electro-taxis (Chen et al., 1997; Pergolizzi et al., 2002; Lee et al., 2005; Gao et al., 2015). Lastly, our evidence suggests that, different from electrotaxis, PKBR-1 acts as a negative regulator during the aerotactic *corona* migration. The different aerotaxis behavior between the *pkbR1*^*null*^ and mTORC2-deficient mutants is puzzling. Besides its mTORC2 independent activation, the molecular mechanisms by which PKBR-1 negatively regulates the *corona* speed remain currently unknown. PKB activation is rather complex, and its full activation is achieved only when both the activation loop (AL) and hydrophobic motif (HM) are phosphorylated (Pearce et al., 2010). However, partial PKB activation can be attained in an mTORC2-independent way through sole AL phosphorylation (Guertin et al., 2006). Strikingly, the role of PKBR-1 seems to be strictly restricted in controlling the velocity of *corona* migration.

**FIGURE 8:**
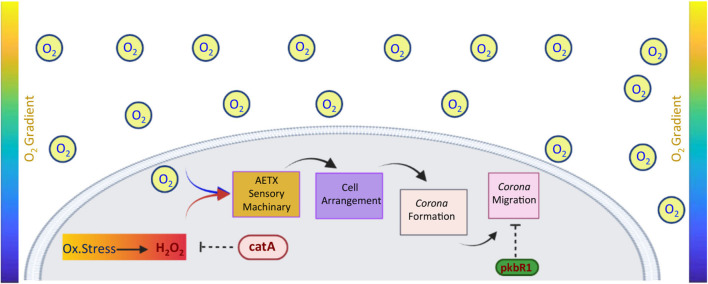
Model of sensing and oriented migration of *Dictyostelium* cells under oxygen gradient. Under oxidative stress conditions, cells accumulate hydrogen peroxide up to a critical threshold (dark red in the rectangle). Once the latter is reached, the cells react and coordinately arrange themselves to give rise to a *corona.* These early events are regulated by unknown aerotaxis (AETX) sensory machinery/ies by which catalase A might represent either a regulatory component or a direct player. Afterward, the *corona* moves toward the higher concentration of oxygen, and its speed is negatively controlled by the PKBR1 protein.

Although the signaling that finely tunes the response to oxygen deprivation in *Dictyostelium* remains mostly unknown, based on our discoveries, we can speculate on a possible model that could be tested in future experiments l (Figure 8). In analogy with mammalian cells, this model proposes that a *Dictyostelium* cell cluster responds to oxygen depletion through a peripheral cell sub-population. This very early event is most likely triggered by an intracellular accumulation of H_2_O_2_ without the involvement of substantial transcriptional events. Afterward, the cell cluster arranges with a peripheral ring structure, the *corona*, that moves outward and whose speed is negatively regulated by PKBR-1.

In conclusion *Dictyostelium*, represents a novel alternative and genetically amenable model that can be widely exploited to unravel some basic cell biology aspects of directional migration toward oxygen.

## Data Availability Statement

The original contributions presented in the study are included in the article/Supplementary Material, further inquiries can be directed to the corresponding author.

## Author Contributions

BP, EB, MO, and MB contributed to the conception and design of the study. BP, EB, CP, and SA conducted the experiments. MB and MO developed the analytic tools and analyzed the data. BP, EB, MO, and MB wrote the first draft of the manuscript. SB revised the manuscript and contributed to the discussion. All authors contributed to manuscript revision, and read and approved the submitted version.

## Conflict of Interest

The authors declare that the research was conducted in the absence of any commercial or financial relationships that could be construed as a potential conflict of interest.

## Publisher’s Note

All claims expressed in this article are solely those of the authors and do not necessarily represent those of their affiliated organizations, or those of the publisher, the editors and the reviewers. Any product that may be evaluated in this article, or claim that may be made by its manufacturer, is not guaranteed or endorsed by the publisher.

## Footnotes

1
www.dictybase.org
