# Supplementary material for: The Dynamics of Aerotaxis in a Simple Eukaryotic Model
Source: Front Cell Dev Biol. 2021 Nov 23;9:720623. doi: 10.3389/fcell.2021.720623 (PMC8650612; doi:10.3389/fcell.2021.720623)
Supplement: Supplementary file 1 [file Data_Sheet_1.docx]

**Supplementary Materials**

**Supplementary Table 1**

**A**

| GENOTYPE | T* [min] | v_i_ [µm/min] | v_f_ [µm/min] |
| --- | --- | --- | --- |
| *WT* | 60 ± 20 | 2.2 ± 0.2 | 0.67 ± 0.14 |
| *GβA^null^* | 49 ± 8 | 2.6 ± 0.1 | ND |
| *HSB1* | 49 ± 9 | 2.3 ± 0.2 | ND |
| *PKBR1^null^* | 38 ± 9 | 3.9 ± 0.8 | 0.51** |
| *catA^null^* | 28 ± 2 | 2.7 ± 0.1 | 0.71** |

**B**

| GENOTYPE | T* [min] | t-statistic | p-value |
| --- | --- | --- | --- |
| *WT* | 60 ± 20 | __________ | __________ |
| *GβA^null^* | 49 ± 8 | 0.67 | 0.53 |
| *HSB1* | 49 ± 9 | 0.64 | 0.55 |
| *PKBR1^null^* | 38 ± 9 | 1.38 | 0.22 |
| *catA^null^* | 28 ± 2 | 2.20 | 0.07 |

**C**

| GENOTYPE | v_i_ [µm/min] | t-statistic | p-value |
| --- | --- | --- | --- |
| *WT* | 2.2 ± 0.2 | __________ | __________ |
| *GβA^null^* | 2.6 ± 0.1 | -3.02 | 0.02 |
| *HSB1* | 2.3 ± 0.2 | -0.78 | 0.47 |
| *PKBR1^null^* | 3.9 ± 0.8 | -4.16 | 0.006 |
| *catA^null^* | 2.7 ± 0.1 | -3.39 | 0.015 |

**Supplementary Table 1: Corona formation time (T*), initial and final velocity (vi, vf) for different D. *discoideum* strains under growing conditions.** **A)** For each strain, we measured T* and v_i_ for three independent experiments, here we report their mean values and their uncertainty (quantified by the standard deviation). **The final velocity of the *corona* (v_f_) of the pkbR1^null^ and catA^null^ mutants were evaluated analyzing only one experiment for each strain; these last two values are compatible with the mean value of v_f_ measured over three independent WT experiments, this result is also shown in Supplementary Figure 7. **B)** T* is defined as the *corona* formation time, *i.e.* the time required for the cluster of cells to constitute a peak of cell density that persistently moves towards higher oxygen concentration. We observed that the discrepancy between the average *corona* formation time of the WT cells and that of the *catA*^null^ mutant exceeded the uncertainty related to their values, thus suggesting that the *catA*^null^ mutant reacted to hypoxia earlier than WT cells. We report the calculated t-statistic and the p-value for the null hypothesis that any two independent samples of T* (WT versus i-^th^ mutant) have identical average values (De Winter, J. C., 2013). **C)** Among the different mutants analyzed the *pkbR1*^null^ cells showed an increased *corona* initial speed (v_i_).

**Supplementary Table 2**

| NAME | GENOTYPE | PHENOTYPE | Reference |
| --- | --- | --- | --- |
| DBS0236389 | pia^G917D^ | abolished aggregation | (Bozzaro et al., 1987; Pergolizzi et al., 2002) |
| DBS0236517 | *lst8^null^* | abolished aggregation | (Lee et al., 2005) |
| DBS0236900 | *ripA^null^* | impaired aggregation | (Lee et al., 2005) |
| DBS0350645 | *piaA^null^* | abolished aggregation | (Chen et al., 1997) |
| DBS0235429 | *AcaA^null^* | abolished aggregation | (Pitt et al., 1992) |
| DBS0236446 | *erkA^-^B^-^* | abolished aggregation | (Nguyen et al., 2010) |
| DBS0236531 - | gpbA^null^ | abolished aggregation | (Lilly et al., 1993) |
| DBS0252655 | *pten ^null^* | abolished aggregation | (Iijima and Devreotes, 2002) |
| DBS0235542 | *pkgB^null^* | impaired aggregation | (Meili et al., 2000) |
| DBS0235525 | *catA*^null^ | abolished catalase activity | (Garcia et al., 2002) |

**Supplementary Table 2.** ***Dictyostelium* strains analyzed**. DB accession numbers, genotype, phenotype and references are listed.

**Supplementary Figure 1**
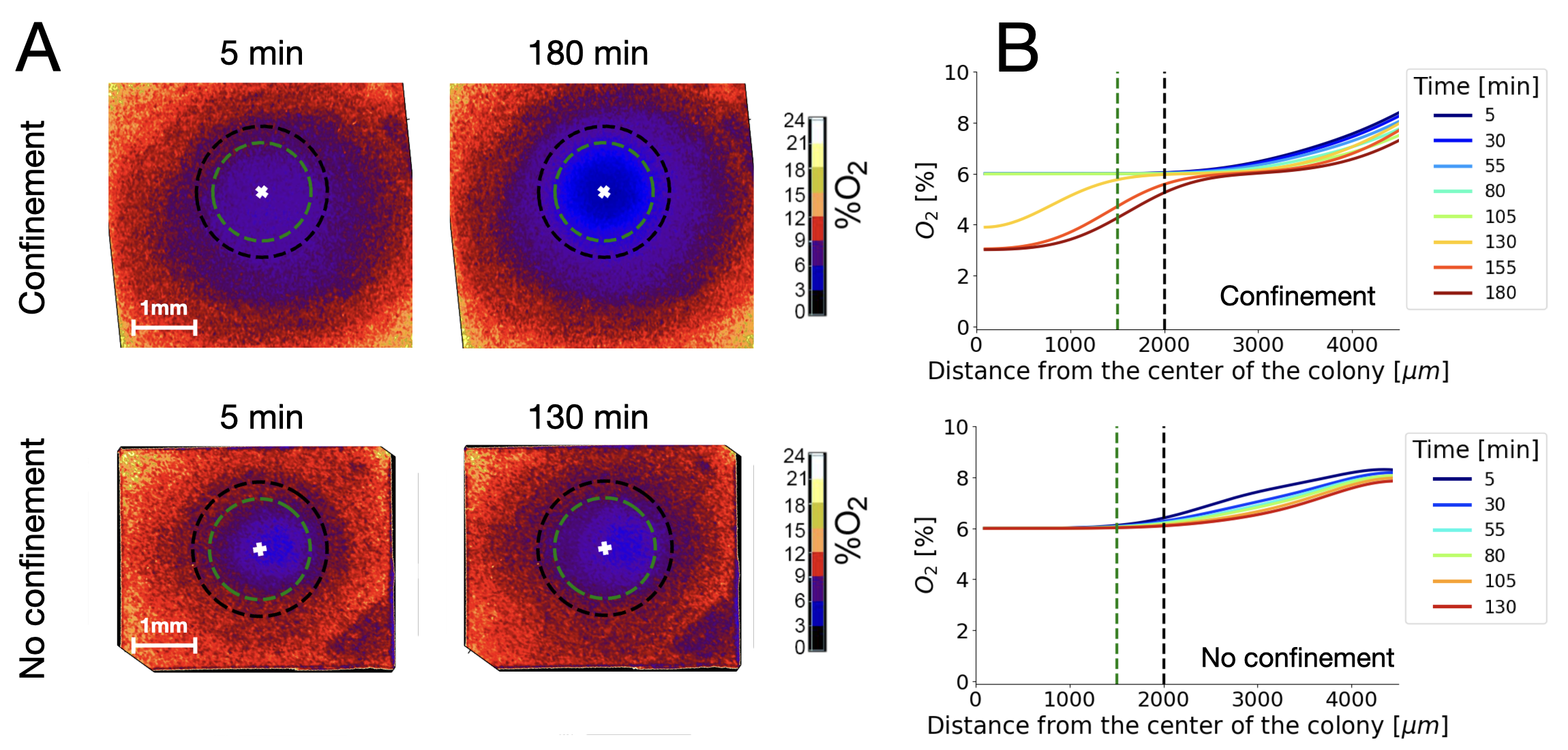


**Supplementary Figure 1 - Oxygen depletion measured during the aerotaxis assay.
A)** Oxygen concentration heatmap measured at the beginning of the experiment and after more than two hours for the confined (top) and unconfined (bottom) colony. These images were obtained using the VisiSens detector unit. **B)** Oxygen concentration profiles in the radial direction at different times. In **A)** and **B)** The typical size of the colony (R = 2000 µm) and the typical radial distance at which the high-density ring was first detected (R = 1500 µm) are marked with black and green dashed lines, respectively.

**Supplementary Figure 2**
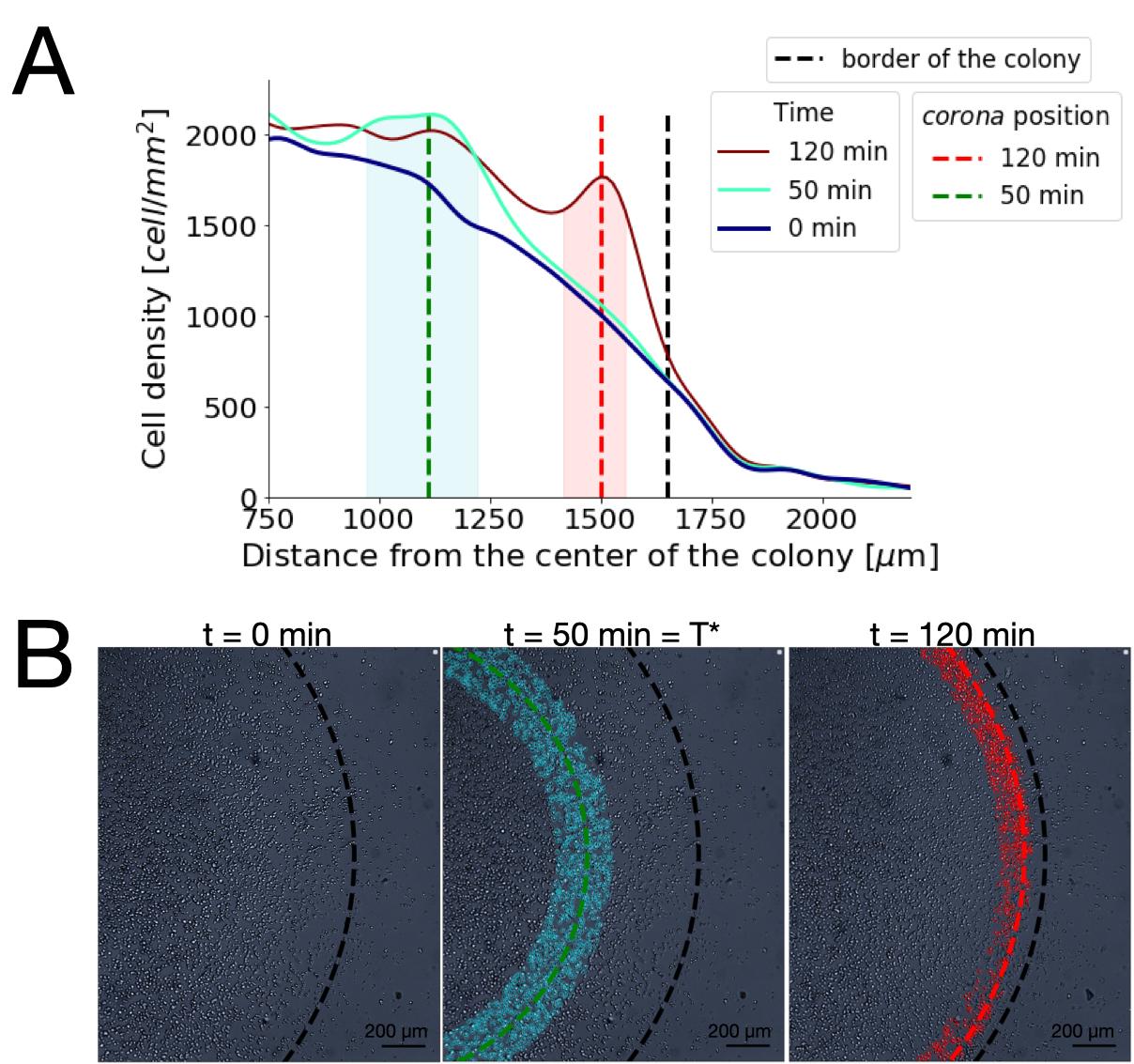


**Supplementary Figure 2. The formation of the *corona* takes place within the border of the growing cell cluster. A)** We report the cell density profiles at different times (0, 50, and 120 min); at the beginning of the experiment (blue profile) the cells were scattered over a radius of approximately 1650 µm, marked by the black dashed line. During this experiment, we detected a peak of density fifty minutes after the confinement of the cells cluster. The radial distance at which the corona was formed is represented by the green dashed line, while the green shaded region represents its width. After its formation, the corona required more than an hour to approach the border of the colony, as shown by the red dashed line that indicates the position of the density maximum at t = 120 min. **B)** We display the raw data (namely the frames) from which we assessed the cell density profiles shown in panel (A), in colors we highlighted the cells of the corona corresponding to the shaded regions of the panel (A). Additionally, we uploaded the complete movie (0-5 hours) of this experiment (Supplementary Movie 2).

**Supplementary Figure 3**
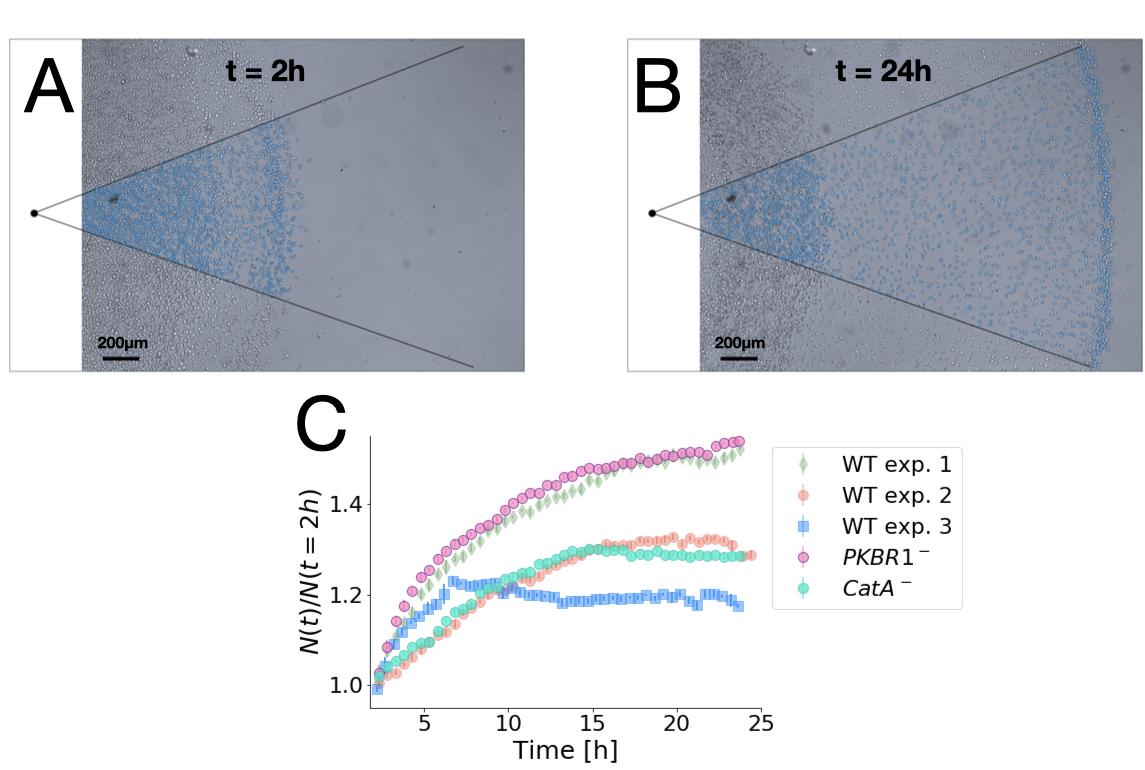


**Supplementary Figure 3. Assessment of cell duplication in the confined system.** Panels **(A)** and **(B)** refer to the same experiment (‘WT exp. 3’) at two different time points (2 hours and 24 hours under confinement). To estimate the role of cell duplication during the collective migration, we considered a circular sector of the growing cells cluster (delimited by the black lines) and the cells localized in it using the Python package Trackpy (blue circles). The increase in the number of cells counted in this specific area can only be attributed to cell duplication, because for symmetry reasons we did not expect any flux of cells through the circular sector. We were interested in the impact of cell duplication on the propagation of the *corona*, therefore we excluded from the range of observation the first 2 hours of the phenomenon just to be sure that the *corona* is completely formed and that it is propagating outside the high-density region. At any time (t), the impact of cell duplication was quantified by the ratio between the current number of cells, N(t), and the number of cells measured 2 hours after the colony confinement. We used this approach to evaluate the role of duplication in different strains, other than WT, **(C)**: overall, in the time range 2-15 hours there was an increase in the ratio N(t)/N(t = 2h). However, after approximately 15 hours, the number of cells was maintained, meaning that duplication is not essential to the propagation of the front**.**

**Supplementary Figure 4**
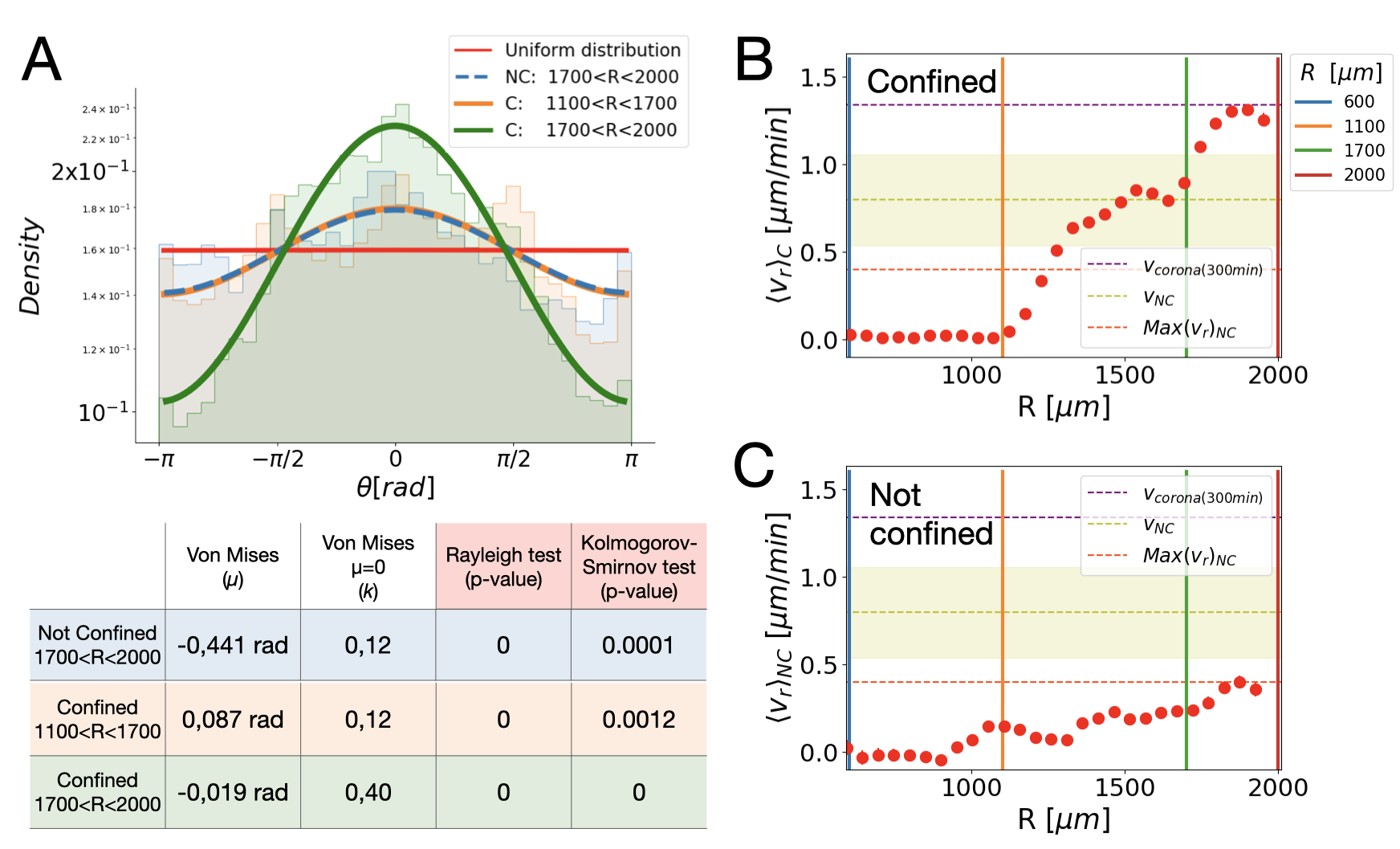


**Supplementary Figure 4. Quantification of the directionality of *Dictyostelium* cells.** **A)** For each event detected with the tracking procedure, we collected the angular displacement θ with respect to the radial direction (*i.e.*, the direction of the oxygen gradient) and the distributions of θ for different groups of cells plotted according to their location: not confined cells located in the outermost region of the cluster (NC: 1700 µm < R < 2000 µm), cells lying in the low-density intermediate region of the confined cluster (C: 1100 µm < R < 1700 µm), and cells belonging to the *corona* (C: 1700 µm < R < 2000 µm). We fitted a Von Mises distribution estimating the mean direction of cells (µ, first column of the table) (Fisher and Annesley 2006). Next, we fitted a Von Mises distribution centered in the radial direction to assess the average accuracy along this orientation (*k*, second column). We performed the Rayleigh test and the Kolmogorov-Smirnov test to confirm the incompatibility between the uniform distribution and the observed distributions of the angular displacement (last columns). We measured the average radial component of the instantaneous velocity 〈v_r_〉 of cells as a function of their radial position for both the confined **(B)** and the not confined condition **(C)**. We report in yellow the velocity of expansion of the colony in the non-confined condition (v_NC_ = 0.8 ± 0.3 µm/min, mean value ± standard deviation), to serve as a benchmark. The orange dashed line highlights the maximum radial velocity measured in the not confined cluster of cells. In figure (B), as expected, for the cells of the *corona* we report an average radial velocity that is compatible with the *corona* propagation speed (v*_corona_* = 1.3 µm/min, purple dashed line).

**Supplementary Figure 5**
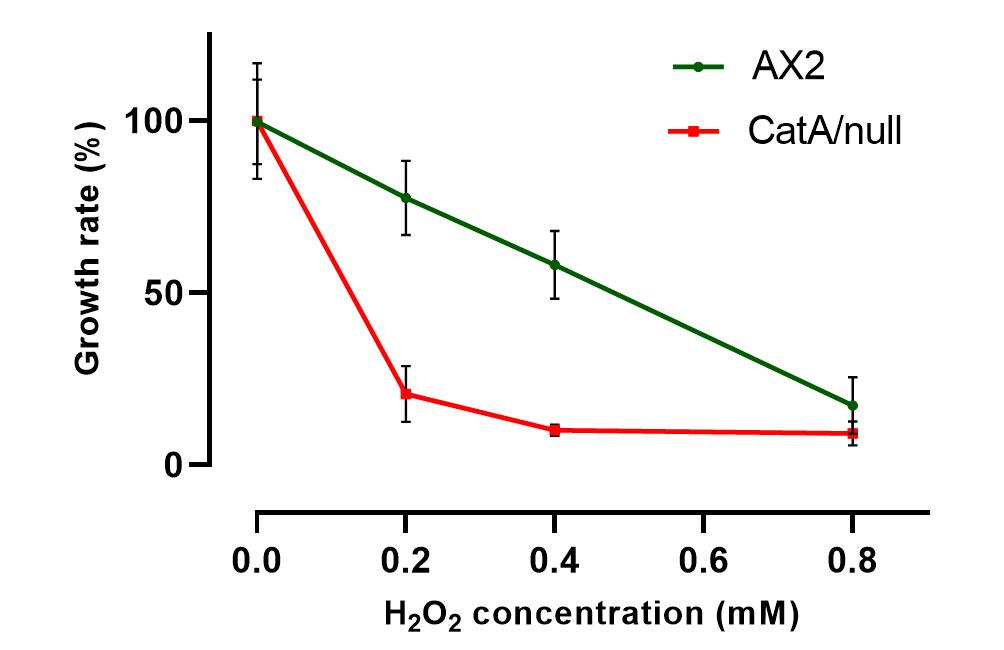


**Supplementary Figure 5. Dose-dependent response of AX2 and *catA^null^* cells to H_2_O_2_.**

Axenically growing AX2 (green line) or *catA^null^* (red line) cells were harvested at exponential phase and seeded in a 96 well plate at a density of approximately 15-20,000 cell/well. Afterwards, hydrogen peroxide (H_2_O_2_) was added to different final concentrations (0.2, 0.4 and 0.8 mM). Cells were then incubated for 48 hrs at 23°C in the dark and eventually counted with the help of a haemocytometer. Data were normalized *vs* the untreated (control) sample and plotted as a mean value + S.D. Each experimental condition was carried-out in technical triplicate and the experiments were repeated at least three times. Line graph representing the mean percent survival relative to untreated cultures. Error bars indicate the standard deviation and were calculated in at least three experiments.

**Supplementary Figure 6**
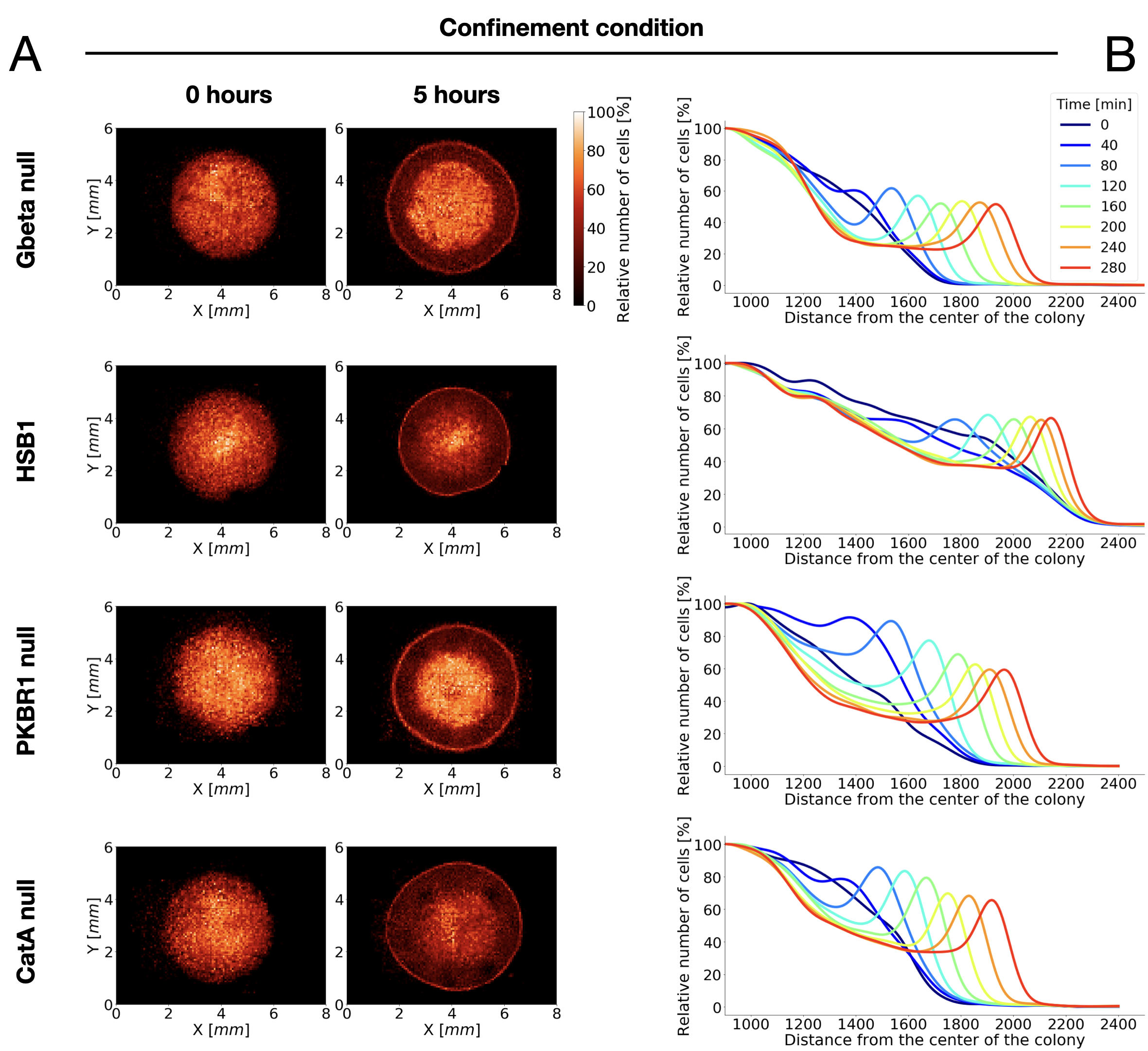


**Supplementary Figure 6**. **The *Dictyostelium* colony mutants react to the oxygen gradient.** **(A)** Distribution of *Dictyostelium* growing cells at the beginning of the experiment and after 5h in the confined conditions. **(B)** Corresponding cell density profiles (estimated as explained in the Materials and Methods) along the radial direction at different times.

**Supplementary Figure 7**
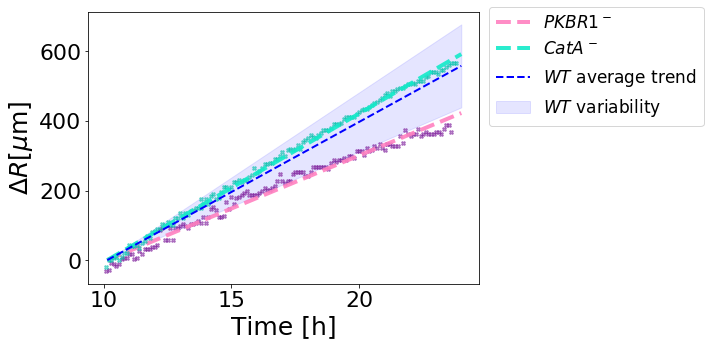


**Supplementary Figure 7. The coordinated migrations of *catA^null^* and *pkbR1^null^* mutants are indistinguishable from those of WT cells within the 10-24 hours time scale**. We evaluated the displacement of the *corona* after ten hours from its formation for growing *catA*^null^ and *pkbR1*^null^ cells: in both cases, the displacement of the *corona* grew linearly over time, and its velocity was similar to the average velocity measured with WT cells and represented in blue (^WT^v_f_ = 0.67 ± 0.14 µm/min, *^pkbR1^*^null^v_f_ = 0.51 µm/min, *^catA^*^null^v_f_ = 0.71 µm/min).

**Supplementary Movie 1**

Aerotactic migration in growing *Dictyostelium* cells in the confined system (0-5 hours).

**Supplementary Movie 2**

Aerotactic migration in growing *Dictyostelium* cells under confinement (0-5 hours). High resolution movie highlights the formation of the thickened circular front of high cell density (*corona*) emerging inside the cell cluster and then persistently moving following the oxygen gradient.

**Supplementary Movie 3**

Aerotactic migration of *Dictyostelium* starving cells under confinement (0-18 hours).

**Supplementary Movie 4**

Aerotactic migration in aggregation competent *Dictyostelium* cells under confinement (0-3 hours).

**References.**

Bozzaro, S., Hagmann, J., Noegel, A., Westphal, M., Calautti, E., and Bogliolo, E. (1987). Cell differentiation in the absence of intracellular and extracellular cyclic AMP pulses in *Dictyostelium discoideum*. *Dev. Biol.* 123**,** 540-548.

Chen, M.Y., Long, Y., and Devreotes, P.N. (1997). A novel cytosolic regulator, Pianissimo, is required for chemoattractant receptor and G protein-mediated activation of the 12 transmembrane domain adenylyl cyclase in *Dictyostelium*. *Genes Devel.* 11**,** 3218-3231.

De Winter, J. C. (2013). Using the Student's t-test with extremely small sample sizes. Practical Assessment, Research, and Evaluation, 18(1), 10.

Fisher, P. R., and Annesley, S. J. (2006) Slug phototaxis, thermotaxis, and spontaneous turning behavior. *Meth. Mol. Biol.* 346, 137-170.

Garcia, M.X.U., Roberts, C., Alexander, H., Stewart, A.M., Harwood, A., Alexander, S., et al. (2002). Methanol and acriflavine resistance in *Dictyostelium* are caused by loss of catalase. *Microbiology (Reading)* 148(Pt 1)**,** 333-340. doi: 10.1099/00221287-148-1-333.

Iijima, M., and Devreotes, P. (2002). Tumor suppressor PTEN mediates sensing of chemoattractant gradients. *Cell* 109**,** 599-610.

Lee, S., Comer, F.I., Sasaki, A., McLeod, I.X., Duong, Y., Okumura, K., et al. (2005). TOR complex 2 integrates cell movement during chemotaxis and signal relay in *Dictyostelium*. *Mol. Biol. Cell* 16**,** 4572-4583.

Lilly, P., Wu, L.J., Welker, D.L., and Devreotes, P.N. (1993). A G-protein beta-subunit is essential for *Dictyostelium* development. *Genes Devel.* 7**,** 986-995.

Meili, R., Ellsworth, C., and Firtel, R.A. (2000). A novel Akt/PKB-related kinase is essential for morphogenesis in *Dictyostelium*. *Curr. Biol.* 10**,** 708-717.

Nguyen, H.N., Raisley, B., and Hadwiger, J.A. (2010). MAP kinases have different functions in *Dictyostelium* G protein-mediated signaling. *Cell Signal* 22(5)**,** 836-847. doi: 10.1016/j.cellsig.2010.01.008.

Pergolizzi, B., Peracino, B., Silverman, J., Ceccarelli, A., Noegel, A., Devreotes, P., et al. (2002). Temperature-sensitive inhibition of development in *Dictyostelium* due to a point mutation in the piaA gene. *Dev. Biol.* 251**,** 18-26.

Pitt, G.S., Milona, N., Borleis, J., Lin, K.C., Reed, R.R., and Devreotes, P.N. (1992). Structurally distinct and stage-specific adenylyl cyclase genes play different roles in *Dictyostelium* development. *Cell* 69**,** 305-315.
